# Ingested Metallic Spool: A Rare Cause of Acquired Tracheoesophageal Fistula

**DOI:** 10.21699/ajcr.v8i1.531

**Published:** 2017-01-05

**Authors:** Imran Hashim, Nabila Talat

**Affiliations:** Department of Pediatric Surgery, The Children’s Hospital and the Institute of Child Health, Lahore

**Keywords:** Foreign body, Metallic spool, Tracheoesophageal fistula, Child

## Abstract

Foreign body (FB) ingestion is a common problem in children. Prolonged impaction of FB in esophagus may result in tracheoesophageal fistula (TEF). A 6-year-old girl presented with progressive dysphagia and recurrent chest infections. No history of FB ingestion was given by parents. Further investigations revealed FB (spool) in cervical esophagus. Patient was successfully managed by surgery through trans-cervical approach.

## CASE REPORT

A 6-year-old girl presented to the pediatric ENT department with fever, cough and respiratory distress for 5 months. She also had progressive dysphagia with infrequent non-bilious vomiting. On examination, she was febrile (1000F), pulse 100/min, and respiratory rate of 30/min. All laboratory investigations were within normal limits. On chest X-ray a metallic foreign body was found (Fig. 1). The child was developmentally normal and neither gave any history of FB ingestion nor did her parents knew about it. On CT scan chest, there were bilateral pulmonary infiltrates due to recurrent chest infections. In addition, there was a visible foreign body with two prongs at the level of upper trachea. Esophago-bronchoscopy was done by ENT department. An impacted old metallic foreign body at cervical esophagus level with lot of granulation tissue and edema was found. It could not be retrieved at endoscopy. A second attempt also failed.

Patient was shifted to our department for surgical removal of the FB. On exploration through cervical approach, a spool was found stuck in the cervical part of the esophagus (Fig. 2). One wheel of the spool was found in the esophagus, which was recovered and the other wheel was found in trachea. After removal of the spool a 4cm rent was noted in the left posterolateral wall of trachea and a similar sized rent was evident in the anterior wall of esophagus. Tracheal and esophageal margins around the rent were refreshed and repaired. A part of deep cervical fascia was harvested and interposed between these repairs. There was significant air leak during the repair, which was controlled by blocking the rent (intermittently blocking the tracheal rent manually with periods of permissive hypercapnia to work under close monitoring of SPO2 and end tidal CO2 by anesthesia team) and manipulating ETT. Postoperatively, the patient was kept on mechanical ventilation for four days. On 8th postoperative day, barium swallow was done which showed no leak of the contrast. She was allowed orally on 9th post-operative day and discharged on 12th post-operative day. At 3-month follow-up, she is doing fine.

**Figure:**
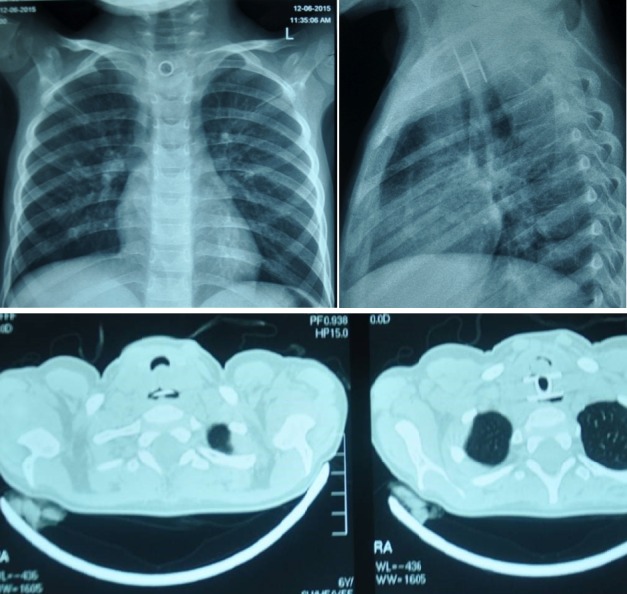
Figure 1: X-ray showing a metallic spool at the thoracic inlet (Upper row). CT scan showing metallic spool with one flange in the esophagus and other in trachea (Lower row).

**Figure:**
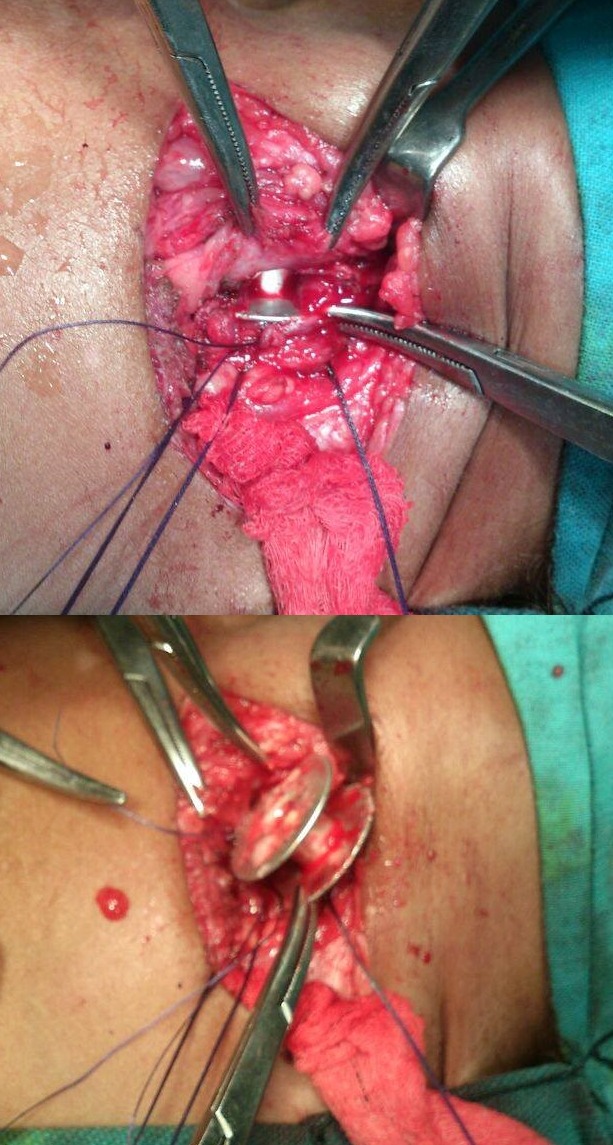
Figure 2: Operative retrieval of sewing machine spool. In the upper part of Figure, one flange of spool has been delivered from opened esophagus, whereas other flange is seen embedded in tracheal wall/lumen.

## DISCUSSION

The presentation of FBs depends on shape, nature, and size as well as site and duration of impaction. FBs which are smooth may pass into intestine without any symptoms while sharp or irregular shape FBs may cause complications such as TEF, esophageal perforation, aorto-esophageal fistula etc. [1-6]. In our case, FB had stuck in esophagus for undocumented time and its sharp flanges eroded posterior esophageal wall and then trachea. The patient had no typical features related to TEF such as chocking with feeds etc. This can be explained by the fact that the spool had an inner hollow, through which esophagus had to communicate with trachea, was completely blocked by granulation tissue.

Endoscopic retrieval is the first-line treatment for FBs impacted in the esophagus. Only 1% need a surgical procedure.[7,8] In our case, multiple attempts were made for endoscopic retrieval but failed. In index case open surgical approach was thus needed. An investment of deep cervical fascia was given between two repairs to avoid recurrence. Sewing machine spool is a common house-hold object and its ingestion may lead to dreadful complications as seen in our patient. Its endoscopic retrieval is also a technical challenge, owing to its shape edges even if attempted early after impaction. A high index of suspicion must be exercised as to presence of FB in un-abating respiratory tract symptoms.

## Footnotes

**Source of Support:** Nil

**Conflict of Interest:** None declared

